# AcneFormer: A Lesion-Aware and Noise-Robust CNN–Transformer for Acne Image Classification

**DOI:** 10.3390/s26082533

**Published:** 2026-04-20

**Authors:** Yongtao Zhou, Kui Zhao

**Affiliations:** School of Cyber Science and Engineering, Sichuan University, Chengdu 610207, China; 2023226240014@stu.scu.edu.cn

**Keywords:** acne classification, medical image processing, convolutional neural network, vision transformer

## Abstract

Convolutional neural networks (CNNs) have been widely used for acne image classification due to their effectiveness in capturing local texture of skin lesions. However, the locality of convolution operations limits their ability to model long-range dependencies. Vision Transformer (ViT) methods address this issue to some extent but their high computational complexity and reliance on large-scale pre-training present challenges. Although CNN–Transformer architecture alleviates this conflict to some extent, acne images present task-specific challenges, including indistinct lesion boundaries, subtle inter-class variations, and various facial interference factors. In this paper, we propose AcneFormer, a lesion-aware and noise-robust CNN–Transformer architecture for acne image classification. We introduce three modules especially for acne tasks: a Lesion Cue Enhancement (LCE) module to highlight discriminative multi-scale spatial patterns, a Cross-Layer Feature Transmission (CLFT) module to enhance cross-layer information flow in Transformers, and a Differential Semantic Denoising (DSD) module to suppress irrelevant responses during deep feature interaction. Extensive experiments show that AcneFormer outperforms several strong baselines. Ablation and external lesion-annotated analyses further show a consistent pattern: LCE mainly improves lesion-sensitive localization and class-balanced recognition, CLFT expands valid cross-depth lesion evidence, and DSD suppresses off-lesion semantic responses.

## 1. Introduction

Acne vulgaris is one of the most common inflammatory skin diseases worldwide and is particularly prevalent among adolescents and young adults [1]. Recent global burden of disease studies indicate that the burden of acne continues to increase among individuals aged 10–24 years [2]. In addition to visible skin lesions, acne can lead to permanent scarring and may impose significant psychological and social burdens on patients; conditions such as permanent scarring can negatively influence self-image and levels of confidence [3,4]. Because treatment selection and therapeutic evaluation rely heavily on the type of lesions and the severity of the disease, accurate classification of acne is a crucial foundation for subsequent clinical management [5]. However, in routine clinical practice, acne assessment still primarily depends on global grading scales and manual lesion counting, which are time-consuming and susceptible to inter-observer variability. Therefore, establishing consistent, objective, and efficient evaluation has become a critical issue that must be addressed.

In recent years, deep learning models have replaced manually designed features and filters and have become the most popular approach in medical image processing [6]. In the classification task of acne images, CNNs remain the primary research direction in this field [7]. Shen et al. were the first to apply CNNs to acne classification, and their results showed that CNNs are effective for acne image analysis, achieving relatively high accuracy across categories [8]. At the same time, however, this work also revealed a structural limitation of such methods: due to the fixed kernel size of CNNs, their receptive fields are limited, making it difficult to cover larger lesions such as cysts and pustules. As a result, the performance on papules, cysts, and pustules was clearly weaker than that on blackheads, whiteheads, and nodules. Since then, numerous improved methods have been developed to address this issue. Some approaches replace coarse sliding-window strategies with explicit segmentation or object detection frameworks [9,10], while others incorporate preprocessing techniques, such as K-means clustering, texture analysis, and HSV-based segmentation, prior to CNN-based classification [11,12]. Additionally, hybrid methods have been developed that combine the representation capability of CNNs with the decision-boundary discrimination ability of support vector machines (SVMs) for lesion recognition [13,14]. Although these methods have achieved certain improvements, their computational costs have become higher, and they still fail to fundamentally solve the limitation of CNN.

Inspired by natural language processing [15,16], Dosovitskiy et al. proposed the Vision Transformer (ViT) for image recognition [17], which models long-range dependencies through a multi-head attention mechanism. Following the great success of Swin Transformer [18], many researchers have applied Transformer architectures to downstream tasks. In the task of acne classification and detection, related works such as AcneDGNet [19] and ETLoViT [20] have also incorporated Transformer mechanisms and achieved promising results. Although Transformer-based methods have an advantage over CNN-based approaches in terms of global modeling, their substantial data requirements remain difficult to overcome. To address this inherent trade-off, some researchers have proposed hybrid CNN–Transformer frameworks for acne image segmentation that aim to reconcile the strengths and limitations of both paradigms [21,22]. Despite this progress, acne image classification still faces several key challenges from the perspective of the task itself. First, diagnostically meaningful visual cues are often small, scattered, and highly scale-varying, and can easily be weakened during feature extraction and fusion, especially when lesion boundaries are ambiguous or lesion scales vary greatly [23,24,25]. Second, reliable lesion-type recognition and downstream acne assessment require the joint utilization of shallow boundary cues, mid-level lesion structural features, and global facial semantic context. However, existing models remain limited in preserving and reusing information across feature hierarchies, a limitation that becomes more pronounced under limited and imbalanced acne datasets [7]. Third, facial acne images often contain substantial interference factors, such as normal skin texture, pores, erythema, pigmentation, and illumination changes, which may dominate deep features and thereby blur the discriminative boundaries between different categories [23,26].

To address these issues, we propose AcneFormer, a hybrid CNN–Transformer network for acne image classification. By introducing the Lesion Cue Enhancement (LCE) module, multi-scale descriptions and coordinate weights are merged into the global modeling process, which improves the directional sensitivity and spatial focus of input features. To address the insufficiency of information preservation and reuse, the Cross-Layer Feature Transmission (CLFT) module expands the original single-channel transmission of the residual stream into four dynamic paths with explicit functional roles, thereby compensating for the insufficient cross-layer bandwidth of the original backbone. At the deeper layer, we introduce the Differential Semantic Denoising (DSD) module to handle the noise present in global semantic interaction at deep stages; it constructs two competitive SoftMax attention maps to cancel nonspecific responses. The main contributions of this work are as follows:

1. To enhance lesion-aware spatial representation, the LCE module is incorporated into the network. By integrating multi-scale channel partitioning with coordinate-aware attention, it preserves fine-grained structural patterns while improving sensitivity to spatially distributed lesion cues during global feature aggregation.
2. Cross-layer information flow is further improved through the proposed CLFT module, which enables dynamic multi-path aggregation across different network depths. This design allows features at different levels to be flexibly reused within each Transformer block, resulting in more comprehensive representation learning.
3. In the deeper stages, semantic representations are refined using the DSD module, where dual attention distributions are constructed and contrasted to suppress non-discriminative responses, thereby improving robustness in global feature interaction.
4. We validate AcneFormer through deployment-matched baseline comparison, repeated-run statistical analysis, class-wise evaluation, failure-case analysis, and external lesion-agreement experiments. Results show that AcneFormer achieves stable and significant performance improvements over multiple strong baselines and the lesion-aware and noise-robust capabilities of AcneFormer have been effectively validated.

The paper is organized as follows: In Section 2, a brief introduction is made to the traditional CNN-based methods and Transformer-based methods used for acne image tasks and advancements in CNN–Transformer hybrid architecture. In Section 3, we first describe the overview of our architecture and then discuss it in detail. Experimental results and evaluation of our method are presented in Section 4. Finally, we conclude in Section 5.

## 2. Related Works

### 2.1. CNN-Based Methods for Acne Image Analysis

Early studies on automated acne analysis mainly relied on convolutional neural networks (CNNs) to model local visual patterns of skin lesions. Some works adopted a region-driven strategy, where candidate acne areas were first extracted through skin detection or color-based segmentation before being classified by CNNs. For example, Shen et al. proposed a pipeline that first distinguishes skin from non-skin regions and then performs multi-class classification on local patches to identify different acne types [8]. Similarly, Yadav et al. utilized HSV-based segmentation and clustering to isolate potential lesion regions prior to CNN-based detection [11]. These approaches reduce background interference but heavily depend on preprocessing quality and may fragment the global facial context.

Subsequent studies attempted to improve acne recognition through deeper end-to-end CNN architectures. Junayed et al. introduced AcneNet, a deep CNN framework for multi-class acne classification that enhances lesion feature extraction through stacked convolutional layers [27]. Islam et al. further proposed a dual integrated deep CNN model that combines multiple convolutional branches and extensive augmentation strategies to improve robustness in seven-class acne recognition [28]. ScarNet extended CNN modeling to acne scar subtype classification, demonstrating the capability of deep convolutional representations for fine-grained dermatological patterns [29]. These works highlight the strength of CNNs in capturing local texture and morphological cues of lesions, but their performance improvements largely rely on deeper networks and feature aggregation.

More recent work incorporates lesion-aware structural designs to better align CNN models with clinical assessment processes. Huynh et al. proposed AcneDet, which first detects multiple lesion types using Faster R-CNN and then predicts acne severity based on lesion counts through a machine learning classifier [12]. Sharmin et al. introduced DLI-Net, which integrates lesion segmentation and classification by combining DeepLabV3 with an Inception-based CNN backbone and employs explainable AI to guide lesion-focused predictions [30]. Qu et al. further developed a coarse-to-fine framework that jointly models lesion counting and acne grading, introducing lightweight refinement modules to enhance fine-grained feature learning [31]. These methods indicate a trend toward incorporating detection, segmentation, and hierarchical decision processes to compensate for the limitations of standard CNN pipelines.

### 2.2. Transformer-Based Methods for Acne Image Analysis

Transformer-based methods provide a complementary line for acne analysis by emphasizing long-range dependency modeling and broader facial context. Early attempts mainly treated the Vision Transformer as a global feature extractor for image-level classification. ETLoViT, for example, employed ViT-B16 to encode facial acne images and then fed the extracted representations into an ensemble of transfer-learning classifiers, showing that attention-based global features can improve acne recognition, while a plain ViT remained much weaker than the final hybrid design on the same task [20]. Subsequent studies moved beyond direct token-based classification toward more structured lesion-aware modeling. Hao et al. used a Swin Transformer to locate lesion regions and further enhanced severity prediction through graph-based feature propagation and label distribution learning on ACNE04, explicitly exploiting correlations in both feature and label spaces [32]. Gao et al. further developed AcneDGNet, which combines a Swin-based feature extractor with lesion detection and lesion-aware severity grading, and validated the framework across smartphone, camera, and VISIA images in both online and offline scenarios [19].

In parallel, Transformer-based encoder–decoder designs were also explored for acne or related skin image segmentation. Junayed et al. proposed a dual Transformer–CNN encoder with feature versatile blocks for acne lesion segmentation and categorization [21], while DermaTransNet showed in related dermatological image analysis that Transformer–U-Net-style architectures can improve region extraction by integrating multi-axis Transformer encoding, attention-mixing decoding, and attentive skip connections [22].

### 2.3. Advancements in CNN–Transformer Architecture

Beyond acne-specific studies, recent advances in general visual recognition have increasingly focused on CNN–Transformer hybrid architectures, aiming to combine the strong local inductive bias of convolution with the long-range dependency modeling capability of attention. Representative works show that this line of research has evolved from simple stage-wise combinations to more tightly coupled and efficient hybrid designs. EdgeNeXt [33] introduced a split depth-wise transpose attention encoder, which integrates depth-wise convolution and channel-wise attention to enlarge the receptive field and encode multi-scale information under lightweight constraints. RepViT [34] further revisited lightweight CNN design from the perspective of Vision Transformers, showing that ViT-inspired structural principles and re-parameterized convolutions can substantially improve the trade-off between accuracy and deployment efficiency on edge devices. These studies suggest that effective local–global collaboration has become a central direction in modern backbone design.

More recent hybrid models have moved a step further from static fusion toward dynamic collaboration between convolution and attention. TransXNet [35] proposed a Dual Dynamic Token Mixer, which splits features into a global attention branch and a local dynamic convolution branch, enabling the network to model long-range context and fine local details simultaneously in an input-dependent manner. Compared with earlier hybrid architectures, such designs better reduce the representation gap between static convolution and self-attention, while maintaining computational efficiency. Nevertheless, existing general-purpose hybrid backbones are not specifically optimized for acne image analysis, where diagnostically important cues are often small, scattered, and highly scale-varying, and where normal skin texture, pores, erythema, pigmentation, and illumination changes may introduce strong interference. Therefore, an acne-oriented hybrid architecture is still needed to further enhance lesion-sensitive feature extraction, cross-layer information interaction, and semantic denoising. This also provides architectural motivation for our proposed method.

Recent attention-enhanced models also suggest three mechanism-level directions that are especially relevant to acne imagery: semantic-aware multi-scale refinement, directional coordinate aggregation, and explicit attention control through sparsity or gating [36,37,38,39]. These trends clarify the design space of AcneFormer more precisely than generic hybridization alone. In our architecture, LCE targets lesion-sensitive multi-scale localization before global aggregation, CLFT targets dynamic reuse of cross-depth lesion evidence, and DSD targets semantic denoising under dense facial contexts.

## 3. Methodology

### 3.1. Overview

**Figure 1:**
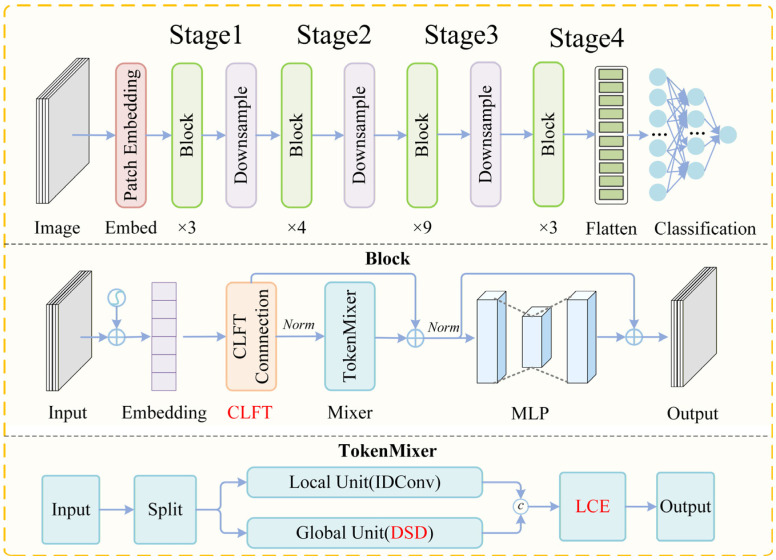
The overview of the AcneFormer.

AcneFormer is a novel neural network designed for acne image classification. As illustrated in Figure 1, the network consists of four stages, where information flow between stages is achieved through downsample operations. The model follows a progressive enhancement strategy. First, an LCE module is introduced in all blocks to enhance the modeling of texture and edge structures. Second, CLFT modules are incorporated in Stage 2, Stage 3, and Stage 4 to expand the bandwidth of cross-layer information transmission. Finally, in Stage 3 and Stage 4, a DSD module is inserted into the latter part of each block to suppress noise in deep semantic interactions.

### 3.2. Backbone Analysis

AcneFormer is built upon TransXNet [35] as the backbone architecture. The key innovation of TransXNet lies in the Dual Dynamic Token Mixer (D-Mixer), which splits the input feature along the channel dimension into two parts. These two parts are processed by Overlapping Spatial Reduction Attention (OSRA) and Input-dependent Depthwise Convolution (IDConv), respectively. In this way, global dependencies and local details can be captured simultaneously. The outputs are then fused using the Squeezed Token Enhancer (STE), enabling efficient integration of receptive fields and inductive biases. Owing to this design, TransXNet provides a highly suitable foundation for further architectural enhancement.

Let the feature entering the D-Mixer be denoted as

(1) $$X\in R^{C\times H\times W}$$

where C, H, and W represent the channel number, height, and width, respectively. The initial feature split can be written as

(2) $$\left[X_{g},X_{l}\right]=Split\left(X\right),{\;\;\;\;\;\;\;X}_{g},X_{l}\in R^{\frac{C}{2}\times H\times W}$$

where $X_{g}$ and $X_{l}$ denote the inputs of the global and local branches, respectively. In the original TransXNet architecture, these two feature streams are processed by OSRA and IDConv, respectively, and then fused through the STE module to produce the final output:

(3) $$\tilde{X}=STE\left(OSRA\left(X_{g}\right),IDConv\left(X_{l}\right)\right)$$

However, from the perspective of the problem addressed in this work, the backbone still leaves room for further enhancement. First, the original global branch lacks an explicit coordinate-sensitive pre-filtering stage before entering OSRA; therefore, edge structures, elongated patterns, and stripe-like regions in high-resolution images may be prematurely averaged during global aggregation. Second, information transmission between blocks still relies primarily on conventional residual connections. Although historical features are preserved, there is no mechanism that enables on-demand feature reading or functional-stream distribution across layers. Finally, once spatial selection and cross-layer reuse are strengthened, the main challenge in deeper stages becomes the over-response of SoftMax attention to background and redundant contexts.

### 3.3. Lesion Cue Enhancement

Following the above motivation, the first issue to address is the spatial filtering of features before the global branch enters OSRA. The objective of this module is to enhance the spatial sensitivity of feature representations so that the model can focus more effectively on region structures associated with acne lesions during subsequent global modeling. Similar direction-aware attention mechanisms have been shown to effectively encode spatial positional information and improve feature representation ability [40]. The LCE module can be divided into two stages: multi-scale channel partitioning and spatial coordinate localization. Unlike conventional coordinate attention or multi-scale designs applied independently, LCE integrates scale-aware decomposition with directional localization before global attention, making it more suitable for lesion-sensitive modeling. Conceptually, LCE is related to semantic-aware multi-scale refinement and directional coordinate attention, both of which aim to preserve boundary-sensitive spatial structure during feature aggregation [37,38]. Unlike these generic mechanisms, LCE couples scale-aware channel partitioning with coordinate localization before the global OSRA branch, making it more directly suited to subtle and spatially scattered facial lesions.

#### 3.3.1. Multi-Scale Channel Partition

Inspired by multi-scale feature decomposition strategies widely used in computer vision tasks to capture structures at different scales [40,41,42], the feature entering the global branch is first divided into multiple scale sub-branches along the channel dimension:

(4) $$X_{g}=\left[X_{g}^{\left(1\right)},X_{g}^{\left(2\right)},\ldots ,X_{g}^{\left(S\right)}\right],X_{g}^{\left(s\right)}\in R^{c_{s}\times H\times W},\sum_{s=1}^{S}c_{s}=C_{g}$$

where S denotes the number of scale branches and $c_{s}$ represents the number of channels assigned to the s scale branch. When the channel number cannot be evenly divided, the remaining channels are sequentially allocated to the first few branches to ensure channel conservation. The key idea behind this design is that different channel groups no longer share the same receptive field. Instead, each group is responsible for describing contextual information on a specific scale.

#### 3.3.2. Coordinate Localization Stage

**Figure 2:**
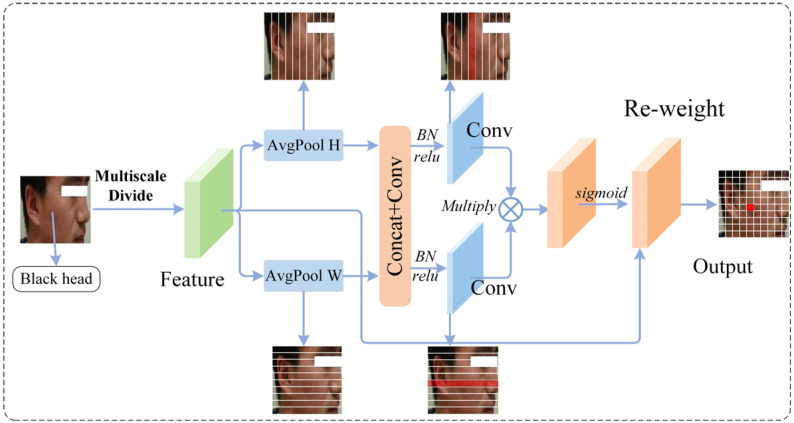
The flow of the coordinate localization stage.

After multi-scale partitioning, the coordinate localization stage is performed. As shown in Figure 2, instead of directly applying two-dimensional global pooling, directional aggregation is conducted along two spatial directions:

(5) $$u_{h}\left(c,h,1\right)=\frac{1}{W}\sum_{w=1}^{W}X_{g}\left(c,h,w\right),\;u_{w}\left(c,1,w\right)=\frac{1}{H}\sum_{h=1}^{H}X_{g}\left(c,h,w\right)$$

where $u_{h}$ denotes the feature aggregated along the height direction and $u_{w}$ denotes the feature aggregated along the width direction. Compared with standard global pooling, these two directional aggregation operations preserve spatial distribution information along different axes. Specifically, aggregation along the width direction captures the variation in responses along the vertical dimension, while aggregation along the height direction reflects response distributions along the horizontal dimension. Unlike traditional global pooling, this directional aggregation strategy preserves key spatial structure information rather than completely collapsing the spatial dimensions.

The resulting features are then passed through a shared transformation layer consisting of Conv–BN–Activation. This operation maps the two directional spatial descriptors into a unified intermediate representation space, enabling the network to learn spatial dependencies within a common representation framework. The intermediate feature is subsequently split into two branches to generate height-direction attention weights $A_{h}$ and width-direction attention weights $A_{w}$. The resulting attention weights are applied to the original feature map [26,43], producing the output of the LCE module:

(6) $$\hat{X_{g}}=X_{g}⊙A_{h}⊙A_{w}$$

where ⊙ denotes element-wise multiplication, $A_{h}$ represents height-direction attention weights, and $A_{w}$ represents width-direction attention weights. Multi-scale descriptors determine which scales to emphasize, while coordinating weights determine which rows and columns to focus on.

For acne image classification, such coordinate-aware mechanisms allow the model to capture local inflammatory regions and their surrounding texture variations more accurately, enabling the global branch to concentrate on visual patterns relevant to lesion recognition.

### 3.4. Cross-Layer Feature Transmission

To improve clarity, we progressively introduce CLFT from conventional residual connections to dynamic multi-path aggregation. In acne image classification tasks, shallow features mainly focus on local textures, color variations, and fine-grained edges, while intermediate features gradually form lesion morphology and regional structures, and deep features capture high-level semantic representations. For acne grading, these three types of information are all indispensable and must cooperate throughout the network depth. For example, when deep semantic representations determine whether a region exhibits inflammatory characteristics, they often still rely on shallow features that describe erythema boundaries, papule textures, and local color abnormalities. Therefore, relying solely on residual connections between adjacent blocks often fails to preserve these cross-layer complementary features. As the network becomes deeper, representations from different layers tend to become increasingly similar, leading to insufficient reuse of historical features.

**Figure 3:**
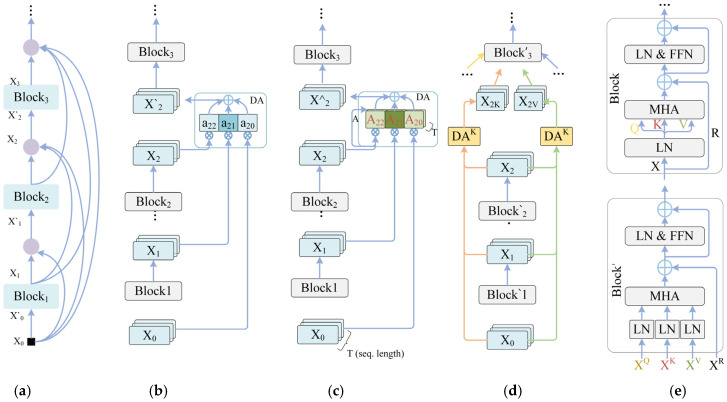
Architecture of the CLFT: (**a**) dense connectivity, (**b**) static dense connection, (**c**) dynamic dense connection, (**d**) Cross-Layer Feature Transmission, (**e**) CLFT in Transformer.

To address this issue, we introduce the CLFT module, which establishes more flexible dynamic feature transmission paths across blocks. Figure 3a illustrates the conventional residual connection mechanism, where each block only receives the output from the previous layer [44]. In contrast, Figure 3b introduces a dense connection structure that allows the current block to access multiple historical feature representations simultaneously, thereby expanding the bandwidth of inter-layer information flow [15]. Although this structure alleviates information attenuation to some extent, the aggregation of historical features still relies on fixed weights, making it difficult for the network to dynamically adjust the importance of different layers based on input content.

CLFT adopts the dynamic dense connection strategy illustrated in Figure 3c. Let the set of historical features accessible to the i-th block be denoted as $\left\{X_{0},X_{1},\ldots ,X_{i}\right\}$. The dynamic aggregation can be expressed as

(7) $${\bar{X}}_{i}=\sum_{j=0}^{i}A_{ij}⊙X_{j}$$

where $X_{j}$ denotes the output of the j-th historical block, $A_{ij}$ represents the dynamic connection weight, and ⊙ denotes element-wise weighting. Unlike static dense connections, these weights are not fixed parameters but are adaptively generated from the input feature of the current block. Specifically, CLFT first employs a lightweight mapping function to generate cross-layer connection weights:

(8) $$A_{ij}=\sigma \left(f\left(X_{i}\right)\right)$$

where f(⋅) denotes a convolutional or linear mapping function and $\sigma \left(\cdot \right)$ denotes the Sigmoid activation.

Through this mechanism, the dynamic weights can adjust the importance of historical features based on the current input, allowing the network to adaptively select more informative feature sources.

Furthermore, as illustrated in Figure 3d, CLFT decomposes Cross-Layer Feature Transmission into multiple functional streams, generating independent cross-layer inputs for the Query, Key, Value, and Residual paths in the attention module. For the m-th functional stream, the cross-layer aggregation can be written as

(9) $$X_{i}^{m}=\sum_{j=0}^{i}A_{ij}^{m}⊙X_{j}$$

where $m\in \left\{Q,K,V,R\right\}$ denotes different functional streams and $A_{ij}^{m}$ denotes the dynamic connection weight for the corresponding stream.

Based on this mechanism, Figure 3e shows the implementation of CLFT in the attention module. Since the Query, Key, Value, and Residual paths originate from different aggregated features, the current block no longer accepts a single input, but instead uses a multi-input structure for attention computation:

(10) $$Y_{i}=\mathrm{Attention}\left(X_{i}^{Q},X_{i}^{K},X_{i}^{V}\right)+X_{i}^{R}$$

where $X_{i}^{Q}$, $X_{i}^{K}$, $X_{i}^{V}$ and $X_{i}^{R}$ represent the four input features obtained through CLFT aggregation respectively. Through this multi-path design, shallow texture information, intermediate structural representations, and deep semantic features can jointly participate in the feature update of the current block.

In summary, CLFT forms a dynamic cross-layer information interaction mechanism within the network, enabling later blocks to simultaneously leverage historical features from multiple depths during global modeling. For acne classification tasks, this Cross-Layer Feature Transmission enhances the joint modeling of lesion textures, inflammatory region structures, and global facial patterns, thereby improving classification performance.

### 3.5. Differential Semantic Denoising

After LCE enhances spatial sensitivity within each block and CLFT establishes dynamic Cross-Layer Feature Transmission, the remaining challenge in deeper stages lies in attention noise during global semantic interaction. In conventional self-attention mechanisms, the SoftMax operation assigns non-zero attention weights to all tokens, causing background regions or redundant contextual information to receive undesired responses. As network depth increases, these non-discriminative activations may accumulate across layers and interfere with the modeling of meaningful semantic patterns.

**Figure 4:**
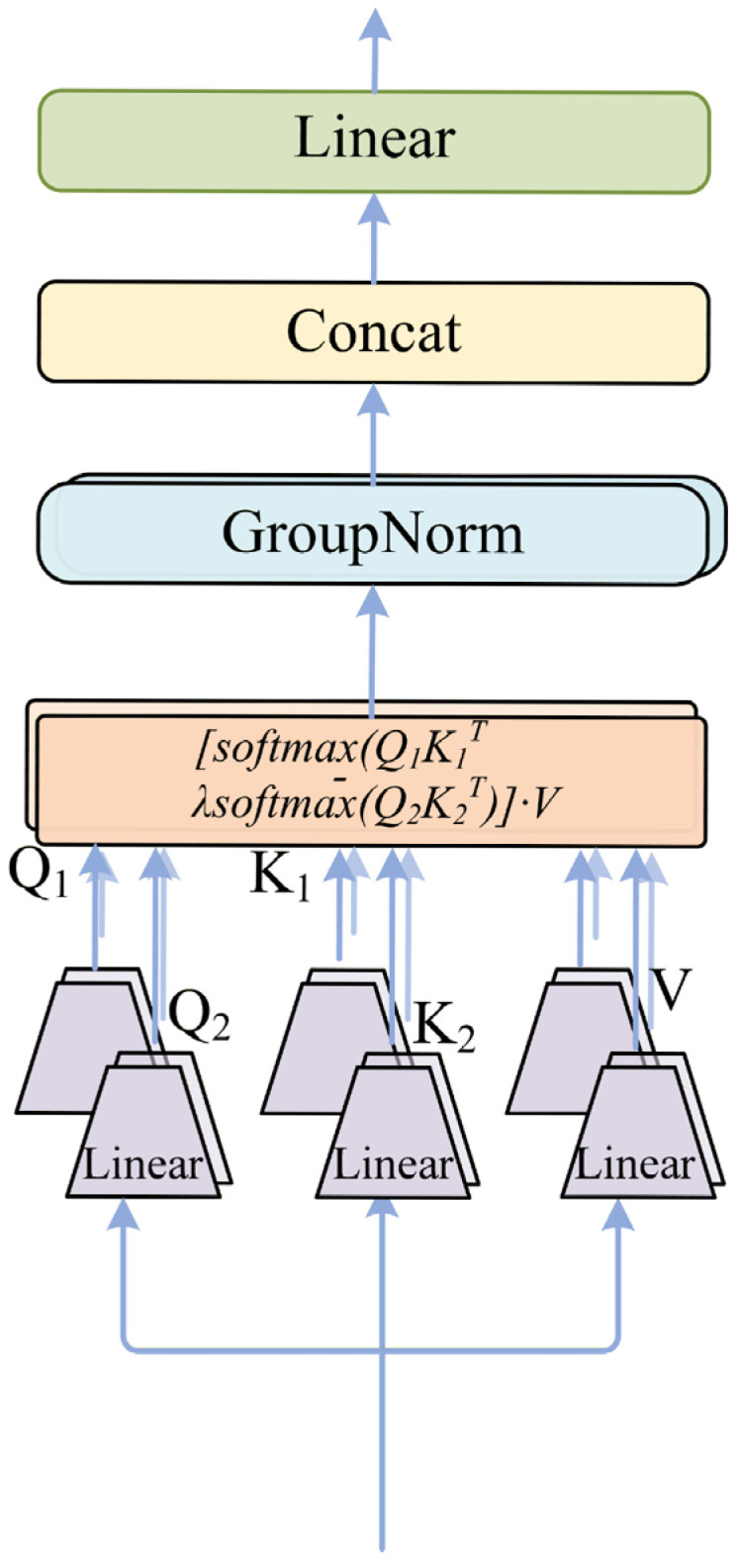
Differential attention mechanism.

To alleviate this issue, we introduce the Differential Semantic Denoising (DSD) module and integrate it into the OSRA Unit, inspired by the differential attention mechanism proposed in Differential Transformer [45]. The core idea is to construct two competing attention maps and suppress nonspecific responses through a differential operation. The overall mechanism is illustrated in Figure 4. DSD should be distinguished from two neighboring attention-control families. Sparse attention methods such as BigBird [39] reduce redundant interactions by constraining the attention pattern, while gated attention mechanisms such as Gated Linear Attention [36] regulate token flow through learned data-dependent gates. Both strategies are valuable, but neither directly constructs a competing semantic distribution that can cancel shared background responses under dense facial contexts. By contrast, DSD preserves dense global interaction and suppresses nonspecific activations through the differential comparison of two SoftMax maps. We therefore position DSD as a semantic denoising mechanism rather than as an efficiency-oriented sparse variant or a single-path gating replacement.

Let the token representation of the deep global branch be

(11) $$X\in R^{N\times d}$$

where N denotes the number of tokens and d denotes the embedding dimension. DSD first projects the input features into two independent Query–Key subspaces through linear transformations:

(12) $$Q_{1}=XW_{q1},K_{1}=XW_{k1},Q_{2}=XW_{q2},K_{2}=XW_{k2}$$

where $W_{q1},W_{k1},W_{q2},W_{k2}\in R^{d\times d_{h}}$ are learnable projection matrices and $d_{h}$ represents the feature dimension of each attention head. In practice, these projections are implemented using linear layers that map the original representations into different semantic subspaces. Meanwhile, the Value representation is obtained through a shared projection:

(13) $$V=XW_{v},W_{v}\in R^{d\times d_{h}}$$

Unlike conventional self-attention that relies on a single correlation map, DSD explicitly models two competing attention distributions. Intuitively, the first attention map captures dominant semantic responses, while the second acts as a competing distribution that approximates background or redundant activations. Based on these projections, two attention maps are constructed:

(14) $$A_{1}=softmax\left(\frac{Q_{1}K_{1}^{T}}{\sqrt{d_{h}}}\right),A_{2}=softmax\left(\frac{Q_{2}K_{2}^{T}}{\sqrt{d_{h}}}\right)$$

The final attention representation is obtained through a differential operation:

(15) $$A=A_{1}-\lambda A_{2}$$

where λ controls the suppression strength of the second attention map. The output of DSD can therefore be written as

(16) Y=AV

This differential formulation allows the model to suppress nonspecific responses automatically. When background tokens receive similar responses in both attention maps, the subtraction operation naturally reduces their influence. Conversely, tokens that appear prominently only in the primary attention map are preserved and further emphasized.

To improve training stability, the suppression coefficient λ is modeled using a reparameterization formulation [46,47]:

(17) $$\lambda =\lambda_{0}+\Delta \lambda$$

where $\lambda_{0}$ is an initialization constant and Δλ is a learnable parameter. This design prevents unstable scaling during early training stages and allows the suppression strength to evolve jointly with the attention projections.

Since differential attention mainly addresses noise in high-level semantic interactions, DSD is deployed only in Stage 3 and Stage 4. In these deeper layers, global contextual relationships dominate feature modeling, and the differential attention mechanism effectively suppresses background interference while enhancing discriminative lesion representations.

## 4. Experiments

### 4.1. Dataset and Metrics

#### 4.1.1. Acne Dataset

Experiments on the primary benchmark are conducted on the Acne Dataset Image dataset, which contains facial acne images grouped into five lesion categories: blackheads, whiteheads, papules, pustules, and cysts. Following the official split used in the current manuscript, as shown in Table 1, 2787 images are used for training, 921 for validation, and 924 for testing. The class distribution is moderately imbalanced: whiteheads account for only 6.5% of all samples, while blackheads account for 26.4%, yielding a max/min class ratio of 4.06. For transparency, class-wise metrics are reported in addition to overall accuracy, UAR, and UF1. Since the public release does not provide structured metadata for acquisition device, illumination protocol, or patient skin tone, we treat this dataset as a lesion-type classification benchmark rather than as a controlled multi-domain clinical cohort. Code will be available at https://github.com/ytyydace/AcneFormer (accessed on 17 March 2026).

#### 4.1.2. External Lesion-Annotated Dataset (ACNE04-v2)

To examine cross-dataset robustness and verify whether the model truly concentrates on lesion regions, we further evaluate the trained models on ACNE04-v2, a publicly released acne image dataset with lesion-level annotations. Because ACNE04-v2 provides lesion locations rather than the same five-class taxonomy as the primary benchmark, it is used here as an external lesion-annotation dataset rather than as a direct five-class classification benchmark. Specifically, we compute CAM–lesion IoU, AILR and BGAR on unseen images to quantify lesion awareness and background suppression under an external data distribution.

#### 4.1.3. Evaluation Metrics

To comprehensively evaluate the performance of the proposed model on the acne lesion classification task, several commonly used metrics are adopted, including accuracy, precision, recall, and F1-score. In addition, considering the potential imbalance among different acne categories, Unweighted Average Recall (UAR) and Unweighted F1-score (UF1) are further employed to provide a more balanced evaluation across all classes. In addition, to quantify the lesion-awareness and noise robustness of the proposed modules on an external lesion-annotated dataset, we report CAM–lesion IoU, attention-in-lesion ratio (AILR), and background activation ratio (BGAR). The formulas for all metrics are shown below:

(18) $$Accuracy=\frac{TP+TN}{TP+TN+FP+FN}$$

(19) $$Precision=\frac{TP}{TP+FP}$$

(20) $$Recall=\frac{TP}{TP+FN}$$

(21) $$F1=\frac{2\times Precision\times Recall}{Precision+Recall}$$

(22) $$UAR=\frac{1}{N}\sum_{i=1}^{N}{Recall}_{i}$$

(23) $$UF1=\frac{1}{N}\sum_{i=1}^{N}{F1}_{i}$$

where TP and TN denote the number of true positive and true negative samples, respectively, while FP and FN represent false positive and false negative samples.

Specifically, given the lesion annotation mask M and the binarized class activation map H, the CAM–lesion IoU is defined as

(24) $$IoU=\frac{\left|H\cap M\right|}{\left|H\cup M\right|}$$

To measure how much attention is concentrated within lesion regions, we compute the attention-in-lesion ratio (AILR) as

(25) $$\mathrm{AILR}=\frac{\sum_{x\in M}A\left(x\right)}{\sum_{x}A\left(x\right)}$$

where A(x) denotes the value of activation at location x.

Finally, to quantify the amount of attention assigned to irrelevant regions, we define the background activation ratio (BGAR) as

(26) $$\mathrm{BGAR}=\frac{\sum_{x\in \bar{M}}A\left(x\right)}{\sum_{x}A\left(x\right)}$$

where $\bar{M}$ denotes the background region. Notably, AILR and BGAR are complementary metrics satisfying AILR+BGAR=1.

### 4.2. Experimental Setup

The proposed methods are implemented based on Pytorch 2.1.0, and all experiments were conducted on an NVIDIA RTX4090 GPU (NVIDIA, Santa Clara, CA, USA) with 24 GB of memory. To evaluate cross-run reliability, the primary experiment was repeated with five random seeds (17, 23, 42, 77, and 102), and the main results are reported as mean ± standard deviation across seeds. In addition, we quantified the statistical significance between AcneFormer and the direct backbone TransXNet using paired *t*-tests on seed-wise accuracy, UAR, and UF1; 95% bootstrap confidence intervals on the mean performance gains; and exact McNemar tests on paired test predictions for each seed. Detailed parameters are presented in Table 2.

### 4.3. Comparison with Representative Baselines and Significance Analysis

#### 4.3.1. Comparison with Baselines

To verify the effectiveness of AcneFormer under a fair capacity regime, we compare it with two acne-specific baselines, Dual-CNN [28] and Weighted-Class [48], as well as three representative attention-enhanced lightweight hybrid backbones, namely EdgeNeXt-Small [33], RepViT [34], and TransXNet [35]. This baseline set is mechanism-oriented rather than only domain-oriented: Dual-CNN and Weighted-Class represent two acne-specific CNN strategies, EdgeNeXt-Small represents efficient multi-scale local–global collaboration, RepViT represents ViT-principled lightweight redesign for deployment-oriented vision backbones, and TransXNet represents dynamic global token mixing and is therefore the most direct backbone comparator.

As shown in Table 3, AcneFormer achieves the best mean performance across all five runs. Compared with TransXNet, AcneFormer improves accuracy from 95.78 ± 0.28% to 97.97 ± 0.27%, UAR from 95.37 ± 0.35% to 97.80 ± 0.34%, and UF1 from 95.88 ± 0.28% to 97.86 ± 0.32%. Bootstrap confidence intervals and McNemar’s test indicate that the improvement over the backbone is stable and statistically significant.

**Figure 5:**
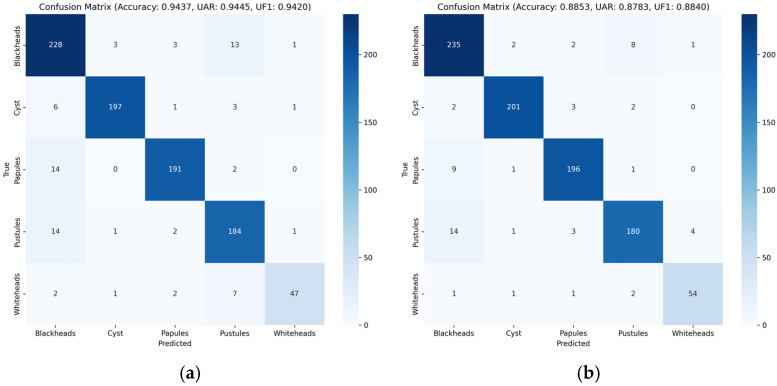

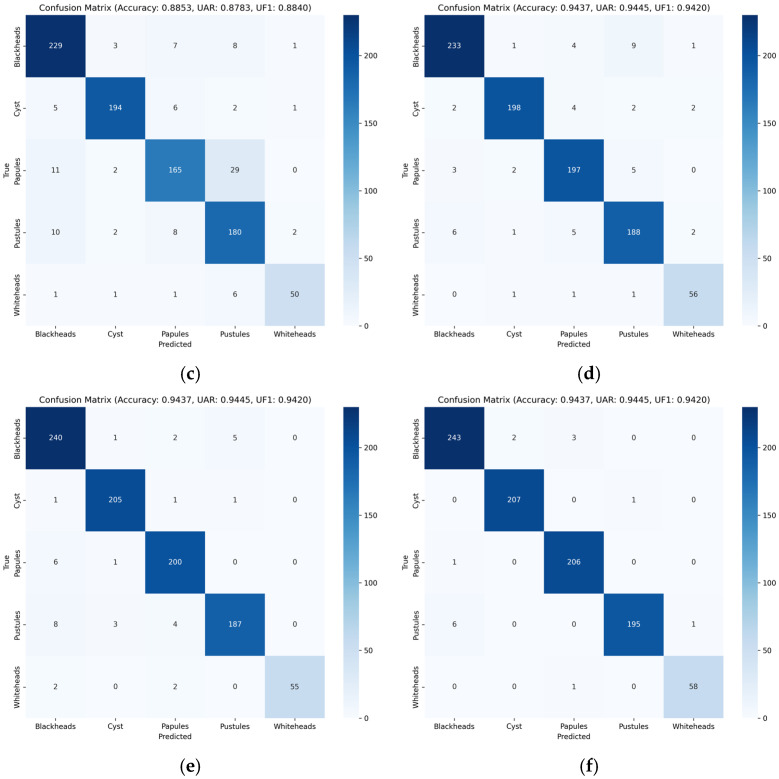
Confusion matrices: (**a**) EdgeNeXt-Small, (**b**) RepViT, (**c**) Weighted-Class, (**d**) Dual-CNN, (**e**) TransXNet, (**f**) AcneFormer (with Seed 42).

The visual evidence in Figure 5 is consistent with the numerical ranking. The two acne-specific baselines in Figure 5c,d still exhibit relatively scattered off-diagonal responses, indicating limited robustness to inter-class similarity and class imbalance. Among the three hybrid backbones, the diagonal entries become progressively more concentrated, from EdgeNeXt-Small in Figure 5a to RepViT in Figure 5b and further to TransXNet in Figure 5e, showing increasingly stronger category discrimination. EdgeNeXt-Small shows obvious confusion between blackheads and pustules, with 13 blackheads misclassified as pustules and 14 pustules misclassified as blackheads, while only 47 whiteheads are correctly classified. RepViT reduces the number of blackheads misclassified as pustules to eight, while pustules misclassified as blackheads remain at 14; meanwhile, the number of correctly classified whiteheads increases to 54. TransXNet further reduces the number of blackheads misclassified as pustules to five and Pustules misclassified as blackheads to eight, while correctly classifying 55 whiteheads. In contrast, AcneFormer in Figure 5f yields the cleanest confusion matrix, with only six pustules misclassified as blackheads and three blackheads misclassified as papules.

**Figure 6:**
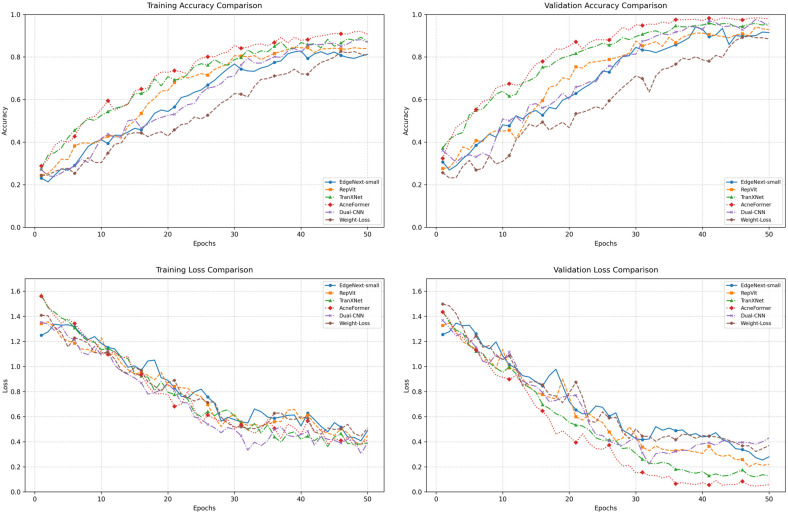
Training and validation performance curves of different baselines and AcneFormer (with Seed 42).

As shown in Figure 6, all models converge stably within 50 epochs, but stronger models reach higher validation-accuracy plateaus and lower validation-loss values. Among the hybrid backbones, the epoch at which validation accuracy first reaches 90% is 39 for EdgeNeXt-Small, 33 for RepViT, 31 for TransXNet, and only 27 for AcneFormer. Meanwhile, the minimum validation loss decreases from 0.25 to 0.19, 0.11, and finally 0.0467. The two acne-specific baselines also converge normally, but their validation trajectories remain less favorable than those of the stronger hybrid models. Therefore, AcneFormer not only achieves the highest final performance, but also exhibits faster convergence and stronger generalization than the compared baselines.

#### 4.3.2. Statistical Significance Between Backbone and AcneFormer

**Figure 7:**
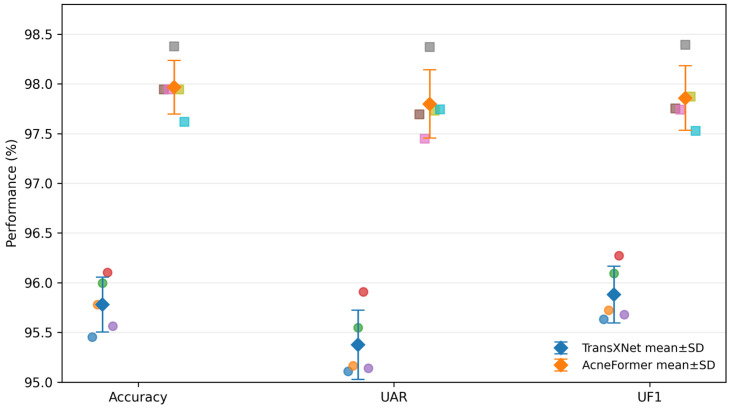
Seed-wise performance and cross-run variance of TransXNet and AcneFormer.

As shown in Table 4 and Figure 7, multi-seed evaluation consistently favors AcneFormer over the direct backbone TransXNet. Across five seeds, AcneFormer achieves 97.97 ± 0.27% accuracy, 97.80 ± 0.34% UAR, and 97.86 ± 0.32% UF1, compared with 95.78 ± 0.28%, 95.37 ± 0.35%, and 95.88 ± 0.28% for TransXNet.

The improvements remain statistically significant under both run-level and sample-level testing. As shown in Table 5 and Table 6, the 95% bootstrap confidence intervals of the mean gains are [1.73, 2.66] for accuracy, [1.86, 3.03] for UAR, and [1.48, 2.50] for UF1, and all three intervals exclude zero. Paired *t*-tests across the five seeds further confirm significant improvements in accuracy $\left(p=4.60\times 10^{-5}\right)$, UAR ($p=1.50\times 10^{-4}$), and UF1 ($p=7.84\times 10^{-5}$). The corresponding mean gains are +2.19, +2.42, and +1.98 percentage points, respectively.

At the paired prediction level, exact McNemar tests are significant under every seed, with *p*-values ranging from $5.65\times 10^{-6}$ to $1.51\times 10^{-3}$. For all five seeds, the number of samples corrected by AcneFormer after TransXNet failed ($n_{01}$ = 22–25) is consistently much larger than the number of samples correctly classified by TransXNet but missed by AcneFormer ($n_{10}$ = 2–5). This pattern indicates that the observed advantage is not caused by random fluctuation in a few isolated cases, but reflects a stable and reliable improvement on paired test samples.

The minority-class whitehead category also shows improved reliability. On the Seed 42 confusion matrices, whitehead recall increases from 93.22% (55/59) for TransXNet to 98.31% (58/59) for AcneFormer. The corresponding 95% Wilson interval shifts from [83.82%, 97.33%] to [91.00%, 99.70%], suggesting that the proposed model substantially reduces missed detections on the rarest class.

### 4.4. Ablation Study and Complexity Analysis

To further verify the contribution of each proposed component, we adopt this progressive order following the architectural design, where spatial enhancement, cross-layer interaction, and semantic denoising are applied in increasing abstraction levels. The results are summarized in Table 7.

As shown in Table 7, regarding mean ± std across five seeds, TransXNet reaches 95.78 ± 0.28% accuracy, 95.37 ± 0.35% UAR, and 95.88 ± 0.28% UF1; +LCE reaches 96.48 ± 0.19%, 96.75 ± 0.20%, and 96.63 ± 0.19%; +LCE + CLFT reaches 97.25 ± 0.20%, 97.35 ± 0.21%, and 97.38 ± 0.19%; and the full model reaches 97.97 ± 0.27%, 97.80 ± 0.34%, and 97.86 ± 0.32%. Therefore, the module-contribution narrative is consistent across both settings: LCE mainly improves lesion localization and class-balanced recognition, CLFT mainly improves cross-depth evidence reuse, and DSD mainly provides the final semantic denoising gain and the best overall performance.

**Figure 8:**
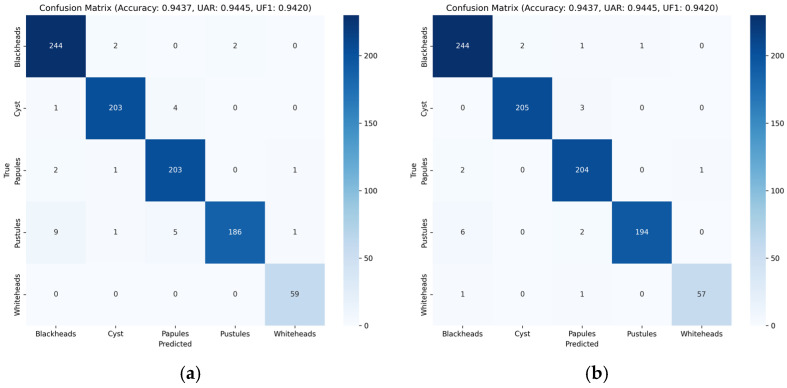
Confusion matrices: (**a**) introduced LCE, (**b**) introduced LCE + CLFT (with Seed 42).

The confusion matrices in Figure 8 provide more fine-grained evidence for the role of each module. After introducing LCE in Figure 8a, the most prominent gain appears in whiteheads, whose recall increases from 93.22% in the baseline to 100.00%, with all 59 samples correctly classified. At the same time, blackhead recall rises to 98.39% and papule recall rises to 98.07%, suggesting that LCE is effective in preserving subtle, scattered, and coordinate-sensitive lesion cues. After further adding CLFT in Figure 8b, the most substantial improvement appears in pustules: the number of correctly classified pustules increases from 186 to 194, while the misclassifications to blackheads and papules are reduced from nine and five to six and two, respectively. This indicates that CLFT is particularly beneficial for inflammatory lesions that require multi-level feature reuse. In the full AcneFormer model shown in Figure 5f, the overall confusion matrix becomes the most compact one. The recalls of cysts and papules both rise to 99.52%; pustules further improve to 96.53%, and whiteheads reach 98.31%. Although the recall of blackheads slightly decreases from 98.39% to 97.98%, the final model achieves the best overall class balance, as reflected by the highest UAR and UF1.

**Figure 9:**
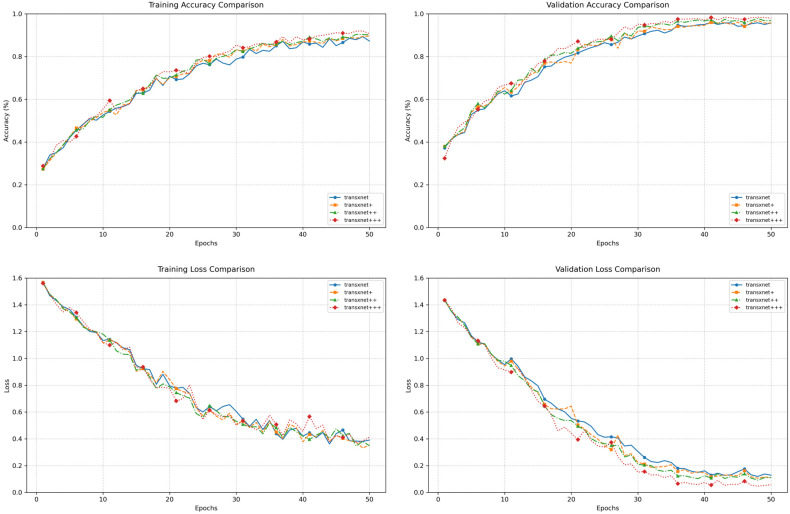
Training and validation performance curves of different ablation models (with Seed 42).

The overall ablation curves in Figure 9 further confirm the progressive effect of the three modules. Since all variants share the same backbone, the curves are closer than those in Figure 6, but the performance ordering remains clear. Validation accuracy reaches 90% progressively earlier, occurring at epoch 31 for TransXNet, epoch 28 for TransXNet + LCE, epoch 28 for TransXNet + LCE + CLFT, and epoch 27 for AcneFormer. More importantly, the minimum validation loss decreases monotonically from 0.11 to 0.10, 0.09, and finally 0.04. This shows that the proposed modules improve not only fitting ability, but also generalization quality and feature robustness, and that their gains accumulate progressively rather than redundantly.

Complexity analysis indicates that the full model introduces moderate rather than prohibitive overhead. As shown in Table 8, compared with TransXNet, AcneFormer increases the parameter count from 12.87 M to 14.95 M and latency from 11.4 ms to 13.2 ms per image, while improving mean accuracy by 2.19 points. This trade-off is acceptable for GPU-based research evaluation and remains a useful reference for later compression or mobile deployment.

### 4.5. Class-Wise Performance of AcneFormer and Cases Analysis

#### 4.5.1. Class-Wise Performance

To further analyze the effectiveness of the proposed model, we conduct a class-wise performance evaluation on the test set. Table 9 reports the detailed precision, recall, and F1-score of both TransXNet and the proposed AcneFormer for each acne category.

As shown in Table 9, AcneFormer consistently outperforms TransXNet across all five acne categories in terms of precision, recall, and F1-score. The improvements are observed for both non-inflammatory and inflammatory lesion classes, indicating that the proposed architecture enhances feature discrimination and class-wise robustness.

In terms of F1-score, AcneFormer improves blackheads from 95.05% to 97.59%, cysts from 98.09% to 99.28%, papules from 96.15% to 98.80%, pustules from 94.68% to 97.99%, and whiteheads from 96.49% to 98.31%. Among these categories, the most notable gains are achieved on pustules and papules, suggesting that AcneFormer is particularly effective for categories with stronger inter-class similarity and relatively more challenging visual patterns.

From the perspective of recall, AcneFormer reaches 97.98% on blackheads, 99.52% on cysts, 99.52% on papules, 96.53% on pustules, and 98.31% on whiteheads, showing improvements over TransXNet in all categories. In particular, the recall gain for whiteheads increases from 93.22% to 98.31%, indicating that the proposed model substantially reduces missed detections for minority classes. In addition, the precision of AcneFormer remains consistently high across all categories, reaching 97.20%, 99.04%, 98.10%, 99.49%, and 98.31% for blackheads, cysts, papules, pustules, and whiteheads, respectively.

Overall, the class-wise evaluation further confirms that AcneFormer provides more balanced and reliable recognition performance than TransXNet. Although slight confusion may remain between visually similar lesion categories, the results demonstrate that the proposed model achieves stronger generalization and better robustness under class imbalance.

#### 4.5.2. Success and Failure Cases Analysis

The strongest successful cases of AcneFormer are those in which the decisive lesion cue is weak at the image level but remains spatially recoverable. This is particularly evident for whiteheads and small papules. In many such images, the discriminative evidence is not a large dominant structure, but rather a combination of tiny bright or reddish micro-patterns, mild boundary elevation, and a limited surrounding halo. A baseline model such as TransXNet can capture part of this information, but its response is more easily diffused across normal facial texture, pores, or broad skin regions after repeated token mixing. In contrast, LCE strengthens multi-scale spatial localization before global aggregation, so the model can preserve fine lesion cues that would otherwise be attenuated. This mechanism explains why the gain is not only reflected in overall accuracy, but is especially visible in the recall improvement for minority or small-structure categories such as whiteheads.

AcneFormer also succeeds more often than the backbone when lesion-like background patterns are present but not dominant. Dark pores, weak pigmentation, mild erythema, and facial shading can all partially resemble acne lesions at low or medium resolution. The DSD module does not create lesion evidence by itself; rather, it reduces the influence of nonspecific semantic responses that compete with true lesions during deep feature interaction. Therefore, in successful cases the final prediction usually emerges from the cooperation of the three modules: LCE improves where to look, CLFT improves what cross-layer evidence is retained, and DSD improves what should be ignored.

Despite the clear overall improvement, the remaining errors are not random; they are concentrated in lesion pairs with genuinely overlapping visual morphology. The most difficult non-inflammatory pair is blackheads versus whiteheads. Both categories are composed of small dot-like lesions with limited surrounding inflammation, and after resizing to 256 × 256 their key differences may occupy only a few pixels. In practice, whether a lesion appears as an open dark comedone or a closed whitish bump can be strongly influenced by illumination, skin oiliness, specular reflection, camera exposure, and viewing angle. As a result, even when the model attends to the correct facial region, the final class decision may still drift between blackheads and whiteheads because the local discriminative evidence itself becomes ambiguous after image compression and global feature mixing.

The main inflammatory confusion is papules versus pustules. Clinically, the distinction often depends on whether a lesion contains a visible purulent white or yellow center in addition to the surrounding erythematous raised region. However, in facial photographs this central cue may be weak, partially occluded, flattened by the viewing angle, or visually blended with neighboring lesions. Conversely, strong specular highlights, surface dryness, or post-inflammatory desquamation may make a papule appear more pustule-like than it really is. Our architecture reduces this confusion because LCE sharpens small local responses and CLFT helps combine them with broader inflammatory context, but it can still fail when the decisive central feature is missing or visually suppressed in the resized image.

Overall, the representative success and failure cases suggest a consistent interpretation of the proposed design. AcneFormer succeeds when the decisive cue is subtle but still spatially recoverable, when dispersed lesion evidence must be integrated across depths, and when background distractions are present but not overwhelming. It is still challenged when the core cue is visually ambiguous, erased by imaging conditions, or contradicted by multiple co-occurring lesion patterns within one crop. Therefore, the remaining errors do not contradict the motivation of LCE, CLFT, and DSD; rather, they delineate the boundary at which lesion-aware localization, cross-layer reuse, and semantic denoising are helpful but not yet sufficient.

### 4.6. External Lesion Agreement and Interpretability

Although ACNE04-v2 does not share the same five-class taxonomy as the primary benchmark, it provides an external and annotation-rich setting to verify the lesion-aware claim. The results in Table 10 show that AcneFormer yields the best CAM–lesion IoU (0.418 ± 0.077) and the highest AILR (0.800 ± 0.069) while reducing BGAR to 0.194 ± 0.043. These results support two complementary conclusions: first, LCE improves spatial concentration on lesion regions; second, DSD suppresses background responses in deeper semantic interactions.

To provide more intuitive evidence for the lesion-aware and noise-robust behavior of the proposed architecture, we add a qualitative heatmap comparison on two representative ACNE04-v2 image/mask pairs. These external results support a consistent three-part interpretation: LCE improves spatial concentration on lesion regions, CLFT further expands the coverage of spatially dispersed valid lesion responses, and DSD suppresses residual background activation in deeper semantic interaction.

**Figure 10:**
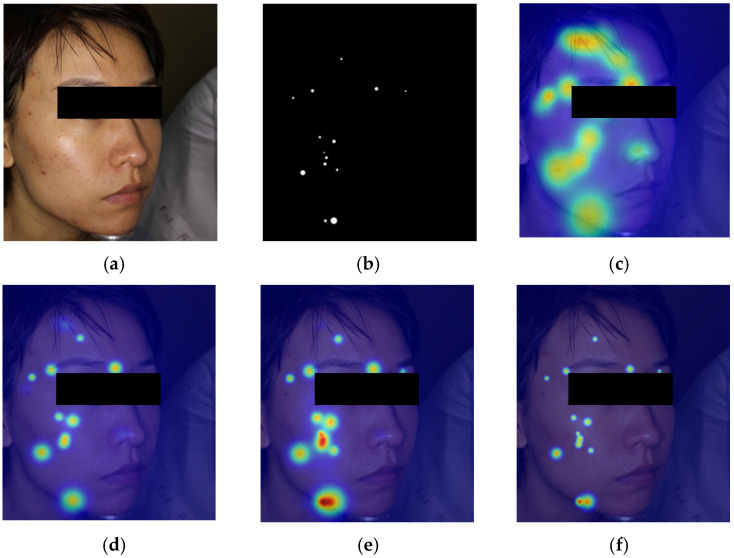
Module-guided qualitative heatmap comparison on Case 1: (**a**) input image, (**b**) lesion mask, (**c**) heatmap of TransXNet, (**d**) heatmap of TransXNet + LCE, (**e**) heatmap of TransXNet + LCE + CLFT, (**f**) heatmap of Acneformer.

As shown in Figure 10, in the baseline panel, TransXNet is shown activating on several forehead and nasal regions while under-covering part of the cheek/chin lesion set. After adding LCE, the broad activation collapses into sharper local hotspots, and the correspondence to valid lesion positions becomes more accurate. After adding CLFT, additional cheek and chin lesions are recovered. In the final AcneFormer panel, most remaining off-lesion responses are removed and the heatmap becomes more compact around the annotated lesion sites.

**Figure 11:**
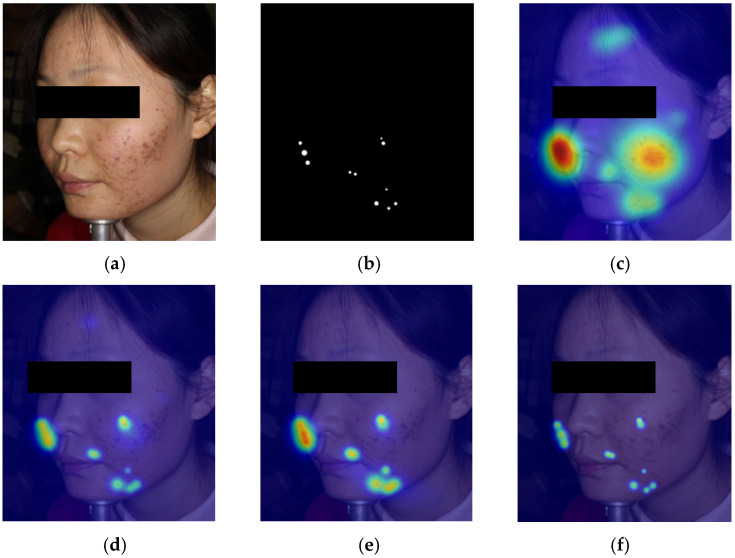
Module-guided qualitative heatmap comparison on Case 2: (**a**) input image, (**b**) lesion mask, (**c**) heatmap of TransXNet, (**d**) heatmap of TransXNet + LCE, (**e**) heatmap of TransXNet + LCE + CLFT, (**f**) heatmap of Acneformer.

In case 2, as shown in Figure 11, TransXNet is shown over-attending to a broad cheek region with lesion-like dark marks and partial nasal-side distractions, while missing part of the distributed lower-cheek/jaw lesion set. After adding LCE, the response becomes more localized, but the number of lesion sites covered is still limited. After adding CLFT, the attention extends to more valid lesion targets along the lower cheek and jaw. In the final AcneFormer panel, the broad cheek noise is strongly suppressed and only compact lesion-aligned responses remain.

The two representative ACNE04-v2 cases provide an interpretable view of the intended role of each module. Specifically, the baseline panels reflect two common failure modes: false activation on lesion-like but non-lesion regions and incomplete coverage of spatially dispersed lesions. The +LCE panels emphasize improved local precision, the +LCE + CLFT panels emphasize the recovery of additional valid lesion sites, and the final AcneFormer panels emphasize the suppression of spurious responses.

## 5. Discussion and Limitations

Despite the strong results, several limitations should be acknowledged. First, the main classification benchmark is a dataset with limited acquisition metadata, which constrains population-level generalization claims. But experiments on external dataset Acne04-V2 alleviate the situation to some extent. Second, CLFT and DSD introduce additional computation, which may matter for real-time mobile deployment. Third, repeated runs and paired statistical testing improve result reliability, but broader subject-disjoint validation across devices, clinical centers, skin tones, and age groups remains necessary. Future work should therefore focus on multi-center external validation, clinically aligned severity assessment, and lightweight deployment-oriented compression of the proposed architecture. We did not include a direct plug-and-play empirical comparison between DSD and sparse- or gated-attention alternatives under the same visual backbone; such controlled comparison remains an important direction for future work.

## 6. Conclusions

In this paper, we proposed AcneFormer, a hybrid CNN–Transformer architecture for facial acne image classification. The proposed framework was motivated by two complementary limitations in existing methods: convolution-based models are effective at capturing local lesion morphology but are less flexible in handling diverse and dynamically varying visual patterns, while Transformer-based models offer stronger global context modeling yet often suffer from weak inductive bias under limited acne data.

To better match the characteristics of acne images, AcneFormer integrates local and global representation learning through three task-oriented components. Across both the single-run ablation and the repeated-run benchmark, these modules exhibit a consistent and complementary contribution pattern. LCE mainly improves lesion localization and class-balanced recognition of subtle categories, CLFT further improves the reuse of complementary cross-depth lesion evidence, and DSD provides the final denoising gain by suppressing off-lesion semantic responses during deep semantic interaction. External lesion-agreement analysis on ACNE04-v2 supports the same interpretation by showing improved lesion concentration together with reduced background activation. Overall, the results suggest that the gains of AcneFormer arise from task-oriented lesion-aware local-global modeling rather than from generic hybridization alone.

## Figures and Tables

**Table 1:** The detailed distribution of samples from Acne Dataset Image.

Class	Train Set	Validation Set	Test Set	Total
Blackheads	735	240	248	1223
Cyst	654	206	208	1068
Papules	621	209	207	1037
Pustules	584	217	202	1003
Whiteheads	193	49	59	301
Total Samples	2787	921	924	4632

**Table 2:** Experimental parameters.

Training Configuration	Parameter
Image size	256 × 256
Epoch	50
Batch size	4
Lr	1 × 10^−4^
Min_lr	1 × 10^−6^
Weight decay	1 × 10^−4^
Optimizer	AdamW
Learning rate schedule	Cosine Annealing
Momentum	0.9
Seed	17, 23, 42, 77, 102

**Table 3:** Repeated-run benchmark (mean ± std).

Model	Accuracy (%)	UAR (%)	UF1 (%)
Dual-CNN	94.21 ± 0.19	94.05 ± 0.28	94.10 ± 0.19
Weighted-Class	88.47 ± 0.44	87.92 ± 0.64	88.09 ± 0.51
EdgeNeXt-Small	91.62 ± 0.38	90.11 ± 0.54	90.87 ± 0.47
RepViT	93.58 ± 0.28	93.18 ± 0.28	93.32 ± 0.28
TransXNet	95.78 ± 0.28	95.37 ± 0.35	95.88 ± 0.28

**Table 4:** Seed-wise performance of TransXNet and AcneFormer.

Seed	Accuracy (TransXNet)	Accuracy (AcneFormer)	UAR (TransXNet)	UAR (AcneFormer)	UF1 (TransXNet)	UF1 (AcneFormer)
17	95.45	97.96	95.11	97.69	95.63	97.75
23	95.78	97.92	95.16	97.45	95.73	97.74
42	96.00	98.38	95.55	98.37	96.09	98.39
77	96.10	97.94	95.91	97.73	96.27	97.87
102	95.56	97.62	95.14	97.74	95.68	97.53
Mean ± SD	95.78 ± 0.28	97.97 ± 0.27	95.37 ± 0.35	97.80 ± 0.34	95.88 ± 0.28	97.86 ± 0.32

**Table 5:** Statistical comparison between TransXNet and AcneFormer based on five-seed paired outputs.

Metric	TransXNet (Mean ± Std)	AcneFormer (Mean ± Std)	Mean Gain (pp)	95% Bootstrap CI (pp)	Paired *t*-Test *p*-Value
Accuracy	95.78 ± 0.28	97.97 ± 0.27	+2.19	[1.73, 2.66]	4.60 × 10^−5^
UAR	95.37 ± 0.35	97.80 ± 0.34	+2.42	[1.86, 3.03]	1.50 × 10^−4^
UF1	95.88 ± 0.28	97.86 ± 0.32	+1.98	[1.48, 2.50]	7.84 × 10^−5^

**Table 6:** Exact McNemar test on paired test predictions for each seed.

Seed	$n_{01}:$ TransXNet Wrong, AcneFormer Right	$n_{10}$: TransXNet Right, AcneFormer Wrong	Exact McNemar *p*-Value
17	25	2	5.65 × 10^−6^
23	22	2	3.59 × 10^−5^
42	24	2	1.05 × 10^−5^
77	22	5	1.51 × 10^−3^
102	22	3	1.57 × 10^−4^

**Table 7:** Ablation study of the proposed modules.

Model	Accuracy (%)	UAR (%)	UF1 (%)
TransXNet	95.78 ± 0.28	95.37 ± 0.35	95.88 ± 0.28
+LCE	96.48 ± 0.19	96.75 ± 0.20	96.63 ± 0.19
+LCE + CLFT	97.25 ± 0.20	97.35 ± 0.21	97.38 ± 0.19
+LCE + CLFT + DSD	97.97 ± 0.27	97.80 ± 0.34	97.86 ± 0.32

**Table 8:** Complexity analysis.

Model	Params (M)	GFLOPs	Latency (ms)	Peak Memory (MB)	Accuracy (%)
EdgeNeXt-Small	5.58	1.32	8.4	1880	91.62
RepViT	8.24	1.63	9.1	1946	93.58
TransXNet	12.87	2.76	11.4	2278	95.78
AcneFormer	14.95	3.31	13.2	2486	97.97

**Table 9:** Class-wise performance of AcneFormer and TransXNet (with Seed 42).

Class	Precision (%) (TransXNet)	Recall (%)	F1 (%)	Precision (%) (AcneFormer)	Recall (%)	F1 (%)	Support
Blackheads	93.39	96.77	95.05	97.20	97.98	97.59	248
Cysts	97.62	98.56	98.09	99.04	99.52	99.28	208
Papules	95.69	96.62	96.15	98.10	99.52	98.80	207
Pustules	96.89	92.57	94.68	99.49	96.53	97.99	202
Whiteheads	100.0	93.22	96.49	98.31	98.31	98.31	59

**Table 10:** External lesion agreement on ACNE04-v2.

Variant	CAM–Lesion IoU	AILR	BGAR
TransXNet	0.307 ± 0.085	0.672 ± 0.054	0.269 ± 0.060
+LCE	0.357 ± 0.082	0.736 ± 0.061	0.239 ± 0.055
+LCE + CLFT	0.377 ± 0.079	0.751 ± 0.065	0.224 ± 0.052
AcneFormer	0.418 ± 0.077	0.800 ± 0.069	0.194 ± 0.043

## Data Availability

The acne image dataset used in this study is publicly available at https://www.kaggle.com/datasets/tiswan14/acne-dataset-image (accessed on 14 January 2026). The Acne04-V2 dataset used in this study is publicly available at https://www.kaggle.com/datasets/karmagames/acne04-v2 (accessed on 11 March 2026). Researchers can freely access and download datasets for academic purposes.

## Abbreviations

The following abbreviations are used in this manuscript:

CNN	Convolutional neural networks
ViT	Vision transformer
LCE	Lesion cue enhancement
CLFT	Cross-layer feature transmission
DSD	Differential semantic denoising
SVM	Support vector machine
OSRA	Overlapping spatial reduction attention
IDConv	Input-dependent depthwise convolution
STE	Squeezed token enhancer
